# Surgical Operations on the Pulp and the Best Means of Extracting It

**Published:** 1878-08

**Authors:** C. G. Edwards


### SURGICAL OPERATIONS ON THE PULP AND THE BEST MEANS OF EXTRACTING IT—REPLANTING AND TRANSPLANTING.

BY DR. C. G. EDWARDS.

*Read before the Kentucky State Dental Association.*

Mr. President and Gentlemen.—As a member of your committee, I respectfully submit a brief and imperfect report on surgical operations on the dental pulp, and the best means of extracting it; also replanting and transplanting teeth.

In making this report I do not claim anything new or original, for no operation of the many perplexing ones in dental science has caused more anxiety to the operator, more pain to the patient, and more research by the earnest men of our profession, than how to treat the dental pulp. Consequently much has been said in dental societies on this subject, and the dental periodicals have sown it broadcast over the land. Therefore, what I will have to say on this trite though still important subject, will be to many of you a repetition of a thrice told tale. Yet, Mr. President, though this report fail to give anything new or interesting, I hope it will lead to a discussion, from which a few of us at least may gain a new idea, learn a new truth.

The subject is a broad one. The question arises, what are surgical operations on the pulp, and at what point do they commence? I will assume that any operation or manipulation which has for its object the salvation or destruction of the pulp when it is threatened or assailed properly comes under this subject. The dental pulp, the most highly organized and exquisitively sensitive tissues of the economy of man, who is so wonderfully and fearfully made, when subjected to the irritating agents resulting from caries, fracture or from whatever source, may cause but trifling inconvenience; but generally gives rise to excruciating pain which may result in serious trouble. How to alleviate the pain, reduce the inflammation, and restore the diseased tissue to a normal condition, or where we do not regard this as practicable, to extract the pulp and nerve successfully, is what we have been striving to learn through long years of toil and research. How sucessful our efforts have been I refer each gentleman present to his individual daily office experience. A few years ago when I assumed the responsibilities of a dental practice, to me this subject was naturally in greater obscurity than now—the anxiety that followed failure in one case, particularly caused me to think and study this subject diligently. My deductions were first in regard to exposed pulps, our efforts should be conservative, our treatment guided and directed by that thorough knowledge of anatomy, physiology, pharmacology and therapeutics, which is so essential to an intelligent consideration of the subject, and upon which succes so entirely depends. Second, whatever operation was decided upon must be conscientiously and thoroughly performed.

I deem it quite useless to occupy your time in referring to the text books and the methods resorted to in years gone by. I will, therefore, proceed at once to the usual, if not universal practice of the day, which is, after removal of all caries that is practical, it being advisable in some instances to leave a portion covering the pulp, rather than entirely expose it. to bathe the cavity and pulp with Creosote, Carbolic acid; Oil of cloves or some similar escharotic, after which, Oxide of zinc made into a paste with one of these agents is placed over the exposed pulp, and then capped with "os. art." This treatment often gives rise to pain more or less severe as the pulp is more or less exposed, and may result in inflammation and death of the pulp. Therefore I have modified the treatment somewhat with very gratifying results in my practice. I never apply Carbolic acid to an exposed pulp. If it is healthy, not inflamed, I want to maintain this condition, and not irritate and inflame it by cauterization. I therefore, merely apply a demulcent, usually phenate of soda or wine of opium, the former being a mild, though reliable styptic, will check the hemorrhage if the pulp should have been wounded. I then prepare a pad of parchment, moisten it in a dilute solution of carbolized camphor, place it over the exposed nerve and fix it there with collodion or solution of gutta percha, then cap with oxy-chloride of zinc. If the pulp is not entirely exposed, I bathe the cavity with carbolized camphor and place some dry calcined oxide of zinc or phosphate of lime on the bottom of the cavity and fill over this with oxy-chloride of zinc; the dry powder will absorb the excess of chloride, thereby preventing its escharotic effect. If the pulp has been subjected to the influence of irritating agents and we find it congested, after removing the cause of irritation, the inflammation may be reduced by soothing applications frequently repeated, such as phenate of soda or trs. opi. and camphor. If, however, we find the pulp ulcerous, I apply carbolized camphor in full strengh, or a solution of the nitrate of silver, subsequently use soothing dressings, and should it return to a normal condition, I proceed to the capping as above described.

The theory advanced by Dr. Cravens for nourishing and protecting exposed pulps, by an application of Lacto Phosphate of lime, is a beautiful one, and not entirely devoid of plausibility, but unfortunately like many other theories, it has as yet no foundation in fact. His claim that the phosphate being digested by the lactic acid is in condition to be absorbed, and is appropriated by the pulp and thereby converted into new bone, is, I think, merely a theory. Although the author and perhaps others may have had beneficial results from its use, I can not think it is either appropriated by the pulps or has any part in the production of new bone. In the hands of your committee it has failed of any good results. The first three and only cases in which it was used, the pain was so extreme, I presume caused by the lactic acid, that it was removed within less than an hour of the application. With these results I had no disposition to make experiments. I will mention one case in which the treatment failed. After removing the "Lacto-Phosphate," and relieving the pain with bi-carbonate soda, it then being almost dark, I hastily placed a piece of spunk saturated with collodion over the pulp and filled with oxy-chloride of zinc, only a momentary pain followed. In one month the excess of oxy-chloride was removed and a permanent filling made. Though eighteen months have elapsed, the tooth is comfortable, and I believe the pulp is alive. I merely mention this as an evidence of the success attending the simplest method of treatment, where failure had resulted from treatment more complicated. As to the best means of extracting pulps, I have but little more to say than, whatever means is resorted to, it should be conscientiously and thoroughly performed. No hinderance, no amount of annoyance or inconvenience should influence us to swerve from our fixed purpose, no part of the operation should be slighted in the least. After devitalizing the pulp, I penetrate it and apply a paste of tanic acid and glycerine or tanic acid and alcohol, which I prefer to remain for several days, when with a delicate instrument thoroughly annealed and slightly barbed or bent at the point, I endeavor by gentle manipulation to detach the pulp and nerve from its bony walls and remove it entire. If this fails, I detach it piece by piece and wash it out with the syringe, and use alcohol freely. If the root is so situated that it can be approached with a drill, I use the flexible spear shaped drill of suitable size and push it to the apex if possible. If the root canal is so shaped as to render access to the apex impossible, I use a solution of tanic acid or salicylic in alcohol allowing it to remain several days, when I may, or may not repeat the application, after which I proceed to fill the canal as is usual. We not unfrequently meet with hypertrophy or fungus growth of the pulp, which some of our text books pronounce incurable and advise extraction of the tooth as the only means of combating the disease. Experience warrants me in the assertion that they yield to treatment more readily than necrosed or partially necrosed pulps. It is quite practicable in many cases to remove it at once, as in the case of a devitalized pulp, for they are rarely more sensitive than gum tissue, Then treat with iodine and carbolic acid or nitrate of silver.

Now, Mr. President, I come to the third and fourth parts of the subject assigned to me. Replanting and transplanting teeth. The first I can make but a meager report of from personal experience—the latter I have absolutely no experience in. Replanting and transplanting you are well aware, was practiced years and years ago, as far back, I believe, as the sixteenth century. And yet replanting has made but little progress up to the present time. Transplanting has made none whatever. The indications suggesting replanting, I think, are few,—few for these reasons: First, but few teeth in a condition suggesting the operation fail to yield to other treatment. Second, but few persons are entirely free from scrofulous taint, scorbutic or gouty diathesis, either of which precludes a reasonable hope of success. The circumstances most favorable for replanting are a good constitution, perfect health and a normal condition of the soft tissues of the mouth, all of which would favor a speedy reunion with but little inflammatian and soreness. It has only been my privilege to see four cases of replanting, one of them is in the mouth of a gentleman who is present with us to-day. This case was one of inflammation resulting from mechanical abrasion. In three days after the operation the tooth was a useful member of the dental arch. Two cases out of three in my practice were successful. One a third inferior bicuspid right side, was extracted by accident, and replaced, immediately the hemorrhage ceased and clot removed, the nerve permitted to remain in the tooth. I never saw this case again, but learned from a member of the family months afterwards the lad sixteen years of age, had not complained of it except for a few days following the operation. The second case was that of a young lady, seventeen years of age, the tooth an inferior left first bicuspid, the root of which was perforated about two-thirds of the distance from the neck to the apex in the effort to enlarge the canal for the more complete removal of the nerve, inflammation setting in and not yielding to treatment extending over several weeks, the tooth was extracted, the cause of trouble discovered, treated and replaced. At the expiration of one week the tooth was almost entirely well, but slightly tender to pressure and not uncomfortably so to percussion.

The third case was a necrosed second superior bicuspid and a very unfavorable one, and resulted in failure. No other treatment was given these cases after replanting, than frequent bathing the soft parts with phenate of soda, which I consider the very best topical application we possess in surgical operations in the oral cavity. I would also suggest the importance of looking to the secretions in the operation of replanting, or any other operation where inflammation is likely to supervene, and if indicated, administer a saline cathartic or an aperient, advise gentle excercise, nourishing diet and total absence of stimulants.

Now, Mr. President, with a few observations on the conditions rendering a correct prognosis uncertain in replanting and transplanting, and I am done. With the almost universal opinion of the medical profession, that but few families are exempt from some constitutional disease, a blood poison, which, in many cases will not tolerate irritating innovations, we should be extremely cautious, lest we attempt it in some case where these conditions exist, and thereby excite a morbid condition, which in a healthy subject, would occasion only simple inflammation, might in a different constitution result in ulceration of a decidedly cancerous type, and in others, might produce fungoid tumors or scrofulous abscess, or our case might have a yet more speedy and tragical termination in trismus.

In conclusion, permit me again to express the wish, and indulge the hope that, what this report fails in, will be more fully elucidated by some of the distinguished gentlemen who honor us with their presence to-day.

